# Increased sodium content after saltwater washing did not compromise the attenuation of blood pressure increase by cod backbone proteins in obese Zucker *fa/fa* rats

**DOI:** 10.1017/jns.2026.10099

**Published:** 2026-05-13

**Authors:** Eira Victoria Rimmen, Åge Oterhals, Tone Aspevik, Maria O’Keeffe, Svein Are Mjøs, Marta Kaminska, Tine Veronica Karlsen, Trude Skogstrand, Alfred Halstensen, Oddrun Anita Gudbrandsen

**Affiliations:** 1 Department of Clinical Medicine, University of Bergen, Norway; 2 Nofima Bergen, Norway; 3 Department of Chemistry, University of Bergen, Norway; 4 Department of Clinical Science, University of Bergen, Norway; 5 Department of Biomedicine, University of Bergen, Norway; 6 K. Halstensen AS, Bekkjarvik, Norway

**Keywords:** Fish proteins, Fish residuals, Hypertension, Obesity, Sodium

## Abstract

A high sodium intake is a major risk factor for raised blood pressure. Consumption of fish fillet is associated with lower blood pressure in humans and other animals, whereas the effects of consuming fish residuals are less explored. To obtain high-quality fishmeal with acceptable sensory properties, the fish residuals may be washed with seawater onboard factory trawlers. This will increase the sodium content in the residuals, and whether this affects blood pressure has not yet been investigated. The primary objective of the present study was to investigate if the increased sodium content in Atlantic cod (*Gadus morhua*) residuals after washing with saltwater affected the development of high blood pressure in male obese Zucker *fa/fa* rats which spontaneously develop hypertension. Rats were fed diets containing cod protein powders prepared from unwashed or saltwater-washed backbone or head fractions (*n* 6/group) as 25% of total protein with the remaining 75% as casein, or casein as the sole protein source (Control group, *n* 6) for six weeks. Blood pressure was measured on day 0 and 40. The diets containing backbone protein powder, independent of whether this fraction was washed with saltwater or not, attenuated the blood pressure increase compared to the Control group, whereas diets containing washed or unwashed head protein powder did not affect the blood pressure development. To conclude, a diet containing cod backbone protein powder attenuated the blood pressure increase in obese Zucker *fa/fa* rats, and this effect was not compromised by the higher sodium content in backbones washed with saltwater.

## Introduction

The prevalence of cardiovascular disease continues to rise, causing an estimated 20.5 million deaths and affecting 598 million people in 2025.^(1)^ High blood pressure is the leading risk factor for cardiovascular and chronic renal diseases,^(2)^ and preventing the development of hypertension to avoid premature cardiovascular disease through a healthy lifestyle, including good dietary choices, is preferable to medical treatment of established hypertension.^(3, 4)^ The evidence for a causal relationship between high dietary sodium intake and raised blood pressure is overwhelming,^(1, 5)^ as the body responds to excess sodium by retaining water, leading to fluid retention and increased fluid volume. The development of salt sensitivity is an important mechanism behind the elevated blood pressure in obese individuals.^(6, 7)^

Consumption of fish as fillets has been associated with lower blood pressure in various populations in both intervention studies and in observational studies.^(8–13)^ Studies in obese Zucker *fa/fa* rats, which are genetically disposed for developing obesity and hypertension,^(14)^ reveal that the development of high blood pressure was delayed when rats were fed diets containing Atlantic salmon muscle,^(15)^ Atlantic cod press cake protein powder containing heads and a high gut content,^(16)^ or hydrolysed press cake protein powder from headed and gutted blue whiting.^(17)^ However, when obese Zucker *fa/fa* rats were fed a diet containing muscle from Atlantic cod, the blood pressure development was not affected.^(16, 18)^ Also, the blood pressure development in spontaneously hypertensive rats was not affected by consumption of hydrolysates produced from cod, haddock or salmon muscles.^(19)^ These studies in rats suggest that residuals such as skin, viscera, heads, and backbones may contain compounds that beneficially affect blood pressure. In support of this, we recently presented evidence that a protein powder containing a mixture of heads, backbones, and skin from Atlantic cod delayed the development of high blood pressure in obese Zucker *fa/fa* rats.^(20)^

The renin-angiotensin-aldosterone system is an important controller of blood pressure by regulating vasoconstriction and reabsorption of sodium and water, where the biologically inactive angiotensin I is converted to the active vasoconstrictor angiotensin II by angiotensin-converting enzyme (ACE).^(21)^ ACE inhibitors are among the most commonly used medications for hypertension treatment^(22)^ and are also prescribed to patients to delay the progression of chronic kidney disease and amend proteinuria.^(23–25)^ Studies have demonstrated an ACE inhibiting *in vitro* activity of cod residuals,^(16)^ headed and gutted blue whiting,^(17)^ and muscles from haddock, cod, and whiting.^(19)^ Motifs with ACE inhibiting activity *in vitro* are identified in both muscle and residuals including skin and backbone from fish,^(26–29)^ thus, a blood pressure-lowering effect after fish intake may be regulated through the angiotensin-renin system.

The fisheries generate large amounts of residuals after filleting, and the majority of the non-utilised volume from the Norwegian seafood industry originates from the demersal fish sector comprising cod, haddock, and flatfish.^(30)^ With proper treatment, these residuals can be used for human consumption as a high-quality protein source. To increase the shelf life and improve the sensory properties of protein powders produced from residuals, it is preferable to wash the residuals to remove undesirable compounds such as haemoglobin which contribute to lipid oxidation and trimethylamine which contributes to a strong fishy smell and taste. On factory trawlers with on-board processing, freshwater is a limited resource. Therefore, the primary objective in the present study was to investigate if washing residuals with saltwater would be an alternative approach as this is likely to increase the sodium content in the residual protein powder and consequently possibly unfavourably affect the blood pressure development in salt-sensitive obese rats that are prone to hypertension. The secondary objectives were to try to elucidate the mechanisms behind any effects of cod residuals on blood pressure development and kidney function in these rats and to investigate the *in vitro* ACE inhibiting potential of the cod residuals. Residual protein powders produced from backbones and heads from Atlantic cod were tested in obese Zucker *fa/fa* rats for six weeks. Grounded backbones and heads were either not washed or washed twice with saltwater. The obese Zucker *fa/fa* rats were chosen as a model since they spontaneously develop obesity and several obesity-related disturbances such as insulin resistance, dyslipidaemia, mild glucose intolerance, and hypertension.^(14)^ The obese Zucker *fa/fa* rats are salt-sensitive and will consequently experience increased blood pressure when fed a high salt diet.^(31)^ These rats also develop albuminuria and focal segmental glomerulosclerosis leading to renal failure.^(32)^ We hypothesised that a diet containing unwashed backbones or heads would attenuate the development of high blood pressure in obese Zucker *fa/fa* rats, and that this effect would be counteracted when the residuals had higher sodium content after being washed with saltwater.

## Methods

### Ethical statement

The study protocol was approved by the National Animal Research Authority (Norway) in compliance with the Animal Welfare Act and the Regulation of animal experiments (Approval No. 29717). All applicable international, national, and institutional guidelines for the care and use of animals were followed.

### Animals and diets

Thirty male obese Zucker *fa/fa* rats (HsdHlr:ZUCKER-Leprfa, from Harlan Laboratories, Indianapolis, IN, USA) were housed in pairs in individually ventilated cages (IVC type 4, blue line from Tecniplast, Buguggiate, VA, Italy). The cages were equipped with plastic housing and were maintained under standard conditions with a temperature of 23–25°C in a room with a controlled light/dark cycle (dark 20:00–06:00), and the cages were placed randomly in the racks. After acclimatisation for a minimum of 7 d under these conditions, the rats were randomly allocated to one of the four experimental groups or the Control group by drawing lots. Rats were given random numbers that could not be linked to the experimental groups. The intervention period started when the rats were 8–9 weeks old and weighed 358 ± 12 g (mean ± SD).

The rats were fed modified semi-purified diets in accordance with the American Institute of Nutrition’s recommendation for growing laboratory rodents (AIN-93G),^(33)^ supplemented with 1.6 g methionine/kg diet as recommended by Reeves^(34)^ for six weeks, and the diets differed only in their protein sources (Table 1). All diets contained 20 wt% of proteins. The rats were in the growth phase throughout the intervention period (based on growth charts for Zucker *fa/fa* rats from Harlan Laboratories, https://www.inotiv.com), therefore, the AIN-93G diet was used instead of the AIN-93 M diet for maintenance containing 15 wt% protein.^(33)^ Also, obese Zucker *fa/fa* rats have an impaired protein metabolism, which leads to inferior protein utilisation and requires a greater protein intake to maintain a maximal rate of protein gain during growth.^(35)^ Casein served as the sole protein source in the Control diet. For the experimental diets, cod residual powder was added to provide 25 wt% of the total dietary protein, while casein constituted the remaining 75 wt% of protein. The ingredients were purchased from Dyets Inc. (Bethlehem, PA, USA) except casein which was purchased from Sigma–Aldrich (Munich, Germany) and the cod protein powders which were prepared by Nofima (Bergen, Norway).

**Table 1.** Composition of the experimental diets

Contents (g/kg diet)	Control diet	Backbone diet	Head diet	Washed backbone diet	Washed head diet
Casein^1^	223.8	167.8	167.8	167.8	167.8
Backbone meal^2^	–	77.9		–	–
Head meal^3^	–	–	85.9	–	–
Washed backbone meal^4^	–	–	–	79.5	–
Washed head meal^5^	–	–	–	–	88.1
Cornstarch	504.1	482.2	474.1	480.5	471.9
Sucrose	90.0	90.0	90.0	90.0	90.0
Cellulose	50.0	50.0	50.0	50.0	50.0
Soybean oil	70.0	70.0	70.0	70.0	70.0
*t*-Butylhydroquinone (TBHQ)	0.014	0.014	0.014	0.014	0.014
Mineral Mix (AIN-93-MX)	35.0	35.0	35.0	35.0	35.0
Vitamin Mix (AIN-93-VX)	10.0	10.0	10.0	10.0	10.0
L-Methionine	1.6	1.6	1.6	1.6	1.6
L-Cystine	3.0	3.0	3.0	3.0	3.0
Choline Bitartrate^6^	2.5	2.5	2.5	2.5	2.5
Growth and maintenance supplement^7^	10.0	10.0	10.0	10.0	10.0

1 contains 89.4% crude protein, 2.4% fat, 10.3% moisture, 2.5% ash

2 contains 64.2% crude protein, 4.6% fat, 4.2% moisture, 25.3% ash

3 contains 58.2% crude protein, 3.7% fat, 4.9% moisture, 29.7% ash

4 contains 62.9% crude protein, 4.2% fat, 4.7% moisture, 29.6% ash

5 contains 56.7% crude protein, 2.7% fat, 5.6% moisture, 34.4% ash

6 contains 41% choline

7 contains vitamin B12 (40 mg/kg) and vitamin K1 (25 mg/kg) mixed with sucrose (995 g/kg) and dextrose (5 g/kg).

### Preparation of cod residual protein powders

Atlantic cod (*Gadus morhua*) was captured in the Norwegian Sea outside Lofoten, Norway, in March 2022. Backbones and heads obtained after land-based filleting operation were frozen before shipment and further processing at Nofima, Bergen. Backbones and heads were partly thawed overnight at approximately 18°C and were milled on a Comitrol 1700 processor (Urchel Laboratories Inc., Valparaiso, IN), vacuum packed in portions of 1–1.5 kg and stored at −23°C until use. The raw materials from cod (backbones and heads) were thawed overnight at 4°C, and were either washed twice with cold saltwater (3.5% NaCl to mimic the salinity in the Norwegian Sea of 35‰, 4°C) or processed as-is. In the washing steps, the raw materials were added saltwater (1:4) and stirred (35 rpm) for 2 min using a helical rod stirrer. The wash water was removed using a coarse sieve, and fine particles in the filtrate collected on a fine sieve (150 µm) and added back to the raw material. The washed backbones and heads were steam-cooked for 15 min and thereafter dried using an air dryer and milled on a Retsch rotomill (aperture 0.75 mm). After two washing cycles, no trimethylamine was detected.^(36)^ The unwashed backbone and head powders were freeze dried and ground as described above. The obtained protein powders were stored at minus 25°C until analysis and formulation of the rat diets.

### Design

Rats were fed ad libitum for six weeks, with free access to tap water and chewing sticks. Rats were weighed weekly. Blood pressure was measured at baseline before the rats were introduced to the experimental diets (day 0) and two days before endpoint (day 40). The blood pressure was measured in conscious rats using the tail-cuff method (described in detail below). The rats were housed individually in metabolic cages (Ancare Corp., NY, USA) on day 30 or 31, for 24 h collection of urine and measurements of water and feed intakes, without fasting in advance. Approximately 1 ml of urine was sampled for analyses before feed was introduced in the metabolic cages (60–90 min), to prevent contamination of the urine. At the end of the intervention period, after a 6 h fast, the rats were euthanised while anaesthetised with isoflurane (Isoba vet, Intervet, Schering-Plough Animal Health, Boxmeer, the Netherlands) mixed with oxygen. Blood was drawn from the heart and collected in BD Vacutainer SST II Advance gel tubes (Becton, Dickinson and Company) for isolation of serum. The right kidney was carefully dissected out and weighed. Lungs were removed and rinsed in saline. Samples of skin, heart muscle, and aorta were harvested and weighed. Serum and tissue samples were frozen at −80°C.

The personnel responsible for handling the rats and performing the analyses were blinded to the rats’ group allocation, and rats were handled and euthanised in random order.

### Analyses of diets

Contents of amino acids and energy in diets, and contents of amino acids, total fat, moisture, and ash in the fish protein powders were measured by Nofima BioLab (Bergen, Norway). Total amino acid composition and content of cysteine + cystine were measured by HPLC after hydrolysis in 6N HCl for 22 h at 110°C, using Waters-Accq-Tag method and fluorescence detection with excitation/emission at 250/395 nm.^(37)^ Tryptophan was chemically determined by the method of Miller.^(38)^ Dietary caloric content was determined by a bomb calorimeter method in accordance with ISO9831:1998^(39)^ using a Parr 6400 calorimeter (Parr Instrument Company, Illinois). Fat content was determined gravimetrically after chloroform/methanol extraction.^(40)^ Moisture content was measured gravimetrically after drying in a forced-air oven at 103 ± 1°C for 4.5 h.^(41)^ Total ash content was determined gravimetrically after incineration at 550°C.^(42)^ Sodium was quantified using inductively coupled plasma optical emission spectrometry. For analysis of fatty acid composition, lipids were extracted from diets by the method of Bligh and Dyer^(40)^ using a mixture of chloroform and methanol. Samples were added heneicosanoic acid (C21:0) as internal standard before methylation. After methylation, lipids in the samples were extracted twice with isooctane. The methyl ester samples were quantified using an Agilent 7890 gas chromatograph equipped with a flame ionisation detector (Agilent Technologies, Inc.) and a BPX-70 capillary column (SGE Analytical Science) as described in Sciotto and Mjøs.^(43)^ Taurine was measured using the Taurine Assay Kit from Cell Biolabs Inc. (cat. no. MET-5071).

### Blood pressure measurements

Rats were pre-warmed in a heating cabinet at 32°C for 30 min before blood pressure was measured using the tail-cuff method (CODA-6, Kent Scientific Corporation, Torrington, CT, USA). Systolic and diastolic blood pressures were measured and mean arterial pressure (MAP) was calculated as (diastolic blood pressure + 1/3 [systolic blood pressure - diastolic blood pressure]). The blood pressure measurements were conducted in conscious rats, and rats were hand-tame and trained to be in the constrainer (Kent Scientific Corporation) before the baseline measurements. The rats were placed in holders on a warming platform (Kent Scientific Corporation). Ten cycles with 5 s delay between cycles (without acclimatisation cycles in advance) were measured under close monitoring by the operator. Readings disturbed by the rats’ change of position, sudden movements or flick of the tail were discarded. The maximum occlusion pressure was set to 250 mmHg, with a deflation time of 15 s and a minimum volume of 15 µl. The same operator performed all blood pressure measurements and was blinded to the rats’ group affiliations. The rats’ blood pressures were measured in randomised order.

### Analyses in rat samples

Serum concentrations of alanine transaminase and aspartate transaminase, both measured with pyridoxal phosphate activation, and urine concentration of creatinine, carbamide, and uric acid were analysed on the Cobas c111 system (Roche Diagnostics GmbH, Mannheim, Germany) using the ALTL (Alanine aminotransferase acc. IFCC), ASTL (Aspartate aminotransferase), CREP2 (Creatinine plus ver.2), UREAL (Urea/BUN), and UA2 (Uric Acid ver.2) kits from Roche Diagnostics. Sodium in urine was quantified using the Cobas c111 system (Roche Diagnostics GmbH, Mannheim, Germany), with the Ion-Selective Electrode module from Roche Diagnostics.

Protein concentration of ACE was measured in serum, lung, and kidney using the Rat Ace (Angiotensin-converting enzyme) ELISA Kit, cat no. EKX-5D437L (Nordic Biosite). Lung and kidney samples were homogenised in PBS, and total protein content was quantified with the Bradford dye-binding method^(44)^ using protein assay dye reagent (Bio-Rad Laboratories, Munich, Germany) with bovine serum albumin (Bio-Rad Protein Assay Standard II, Bio-Rad Laboratories, Hercules, CA, USA) as the standard. Plates were read on a Multiscan FC (Thermo Scientific). The tissue protein concentrations of ACE are presented relative to total protein content.

ACE activity was measured in serum. Samples were incubated for 10 min at 37°C in reaction buffer (PBS with 10 µM ZnCl_2_, and 10 µM lisinopril was added to negative control samples) and added 80 µl of ACE1 substrates to a final concentration of 30 µM. Serum ACE1 activity was measured using one of three substrates: ABZ-FR-K(DNP)-P-OH (pan-domain activity, cat. no. HY-P1853, Chemsupport), ABZ-LFK(DNP)-OH trifluoroacetate salt (C-domain activity, cat. no. A5855, Merck Life Science), and ABZ-SDK(DNP)P-OH trifluoroacetate salt (N-domain activity, cat. no. A5730, Merck Life Science). Fluorescence signal was read every 45 s for one hour using a microplate reader (BioTek Synergy H1, Agilent) at 320 nm excitation and 420 nm emission.

Serum taurine concentration was measured using the Taurine Assay Kit from Cell Biolabs Inc. (cat. no. MET-5071).

Urine concentrations of T cell immunoglobulin mucin-1 (TIM-1) and cystatin C were quantified using the Rat TIM-1/KIM-1/HAVCR Quantikine ELISA kit (cat. no. RKM100) and the Mouse/Rat Cystatin C Quantikine ELISA (cat. no. MSCTC0) from R&D Systems, Bio-Techne, Minneapolis, MN, USA. Urine albumin was quantified using the Rat ALB (urine albumin) ELISA Kit, cat. no. EKX-DAHMVQ from Nordic Biosite. Serum N-terminal Prohormone Brain Natriuretic Peptide (NT-proBNP) was quantified using the Rat NT-proBNP (Sandwich ELISA) ELISA Kit, cat. no. LS-F23593, from LifeSpan BioSciences, Inc. Nitric oxide in urine was measured using the Nitric Oxide Assay Kit (Colorimetric) cat. no. ab65328 from abcam; nitrate is converted to nitrite by adding nitrate reductase, and nitrite is reduced to nitrogen oxide using Griess Reagent I. Griess Reagent II is added to react with nitric oxide to form a stable azo compound. Urine was filtered (Amicon Ultra-0.5 Centrifugal Filter Unit with Ultracel membrane 10K device, Merck KGaA, Darmstadt, Germany) to remove proteins before analysis of nitrite and nitrate. Plates for TIM-1, Cystatin C, albumin, NT-proBNP, and nitrate+nitrite were read on a SpectraMax Plus384 Microplate Reader. All samples were analysed simultaneously in the same plate for each of the assays.

Sodium contents in tissues were analysed as previously described.^(45)^ In brief, samples of skin, heart muscle, and aorta were dried at 50°C, and dry samples were eluted in ultrapure water. Sodium was quantified by ion chromatography, using a Dionex IonPac CS Reagent-Free Ion Chromatography analytical column (P/N 088582) and guard (P/N 088583) on the Dionex Integrion HPLC System equipped with a Cation Dionex Regenerated Suppressor 600 Cation Electrolytic Suppressor and a high-pressure Eluent Generation Cartridge 500 methanesulfonic acid (Thermo Fisher Scientific, Waltham, MA, USA).

### ACE in vitro assay

The dried cod powders were added Trizma buffer (50 mM, pH 8.0, all ingredients from Sigma) and were hydrolysed using trypsin from bovine pancreas (T1426 from Sigma) at 45°C for 4 h.^(46)^ The hydrolysates were heated to 90°C for 20 min to inactivate trypsin and any endogenous ACE. Next, samples were cooled in ice water for 30 min and frozen at −20°C until analyses. Protein content in the hydrolysates were quantified on the Cobas c111 system (Roche Diagnostics GmbH, Mannheim, Germany) using the TP2 (Total Protein Gen.2 monochromatic) kit from Roche. ACE inhibition was measured using the method by Shalaby et al.,^(46)^ using ACE from rabbit lung (A6778-.25UN from Sigma) and 0.88 mM furanacroloyl-Phe-Glu-Glu (F7131 from Sigma) as substrate (dissolved in Trizma buffer; 50 mM, pH 7.5 with 0.3 M NaCl, all ingredients from Sigma). Immediately before reading, 10 μl ACE, 10 μl protein hydrolysate (or ACE inhibitor captopril as positive control), and 150 μl substrate were added to a 96-well polystyrene microtitre plate (Corning®, CoStar®). Samples were run in duplicates. Plates were read at 340 nm every 30th sec for 30 min on a SpectraMax Plus384 Microplate Reader (Molecular Devices) at 37°C. The curves were linear for all samples between 10 and 25 min, and this area was used for calculations. Inter-assay CV was 13.9%, and the mean within-plate CV was 4.1%.

### Outcomes

The primary outcome was to investigate the effects of six weeks intervention with a diet containing saltwater-washed or unwashed proteins from heads or backbones from Atlantic cod on the development of high blood pressure in obese Zucker *fa/fa* rats. The secondary outcomes were to investigate any effects of the diets on serum ACE concentration and activity as well as lung ACE concentration, markers of kidney function and organ damage, and to examine the *in vitro* ACE inhibiting activities of the cod backbone and head protein powders.

### Sample size

The present study is, to the best of our knowledge, the first study to compare the effects of diets containing washed or unwashed protein fractions from heads or backbones from Atlantic cod on the development of high blood pressure in obese Zucker *fa/fa* rats. Therefore, data on effect size were not available for a relevant sample size calculation or minimally detectable effect sizes for the present study. The study was designed with six rats per group based on studies using diets containing proteins from fish on blood pressure development in obese Zucker *fa/fa* rats showing significant effects with group sizes of six rats.^(15–17, 20)^

### Statistical analyses

Statistical analyses were conducted using SPSS Statistics version 28 (SPSS, Inc., IBM Company, Armonk, NY, USA). The experimental groups were compared using one-way ANOVA, with the cut off value for statistical significance set at a probability of 0.05. The present study is regarded as an exploratory study without the possibility of a proper calculation of the necessary sample size, therefore, when appropriate, the ANOVA analyses were followed by Fisher’s LSD post hoc test as recommended by Lee et al.^(47)^ The Fisher’s LSD post hoc test does not adjust the alpha level for multiple comparisons and therefore maintains higher power to detect differences and is less likely to produce Type II errors (false negatives), which is advantageous when finding true effects is prioritised over avoiding false positives. Changes in MAP from baseline to endpoint within each group were tested using the Paired-samples T test, and the within-group changes were compared between the groups using one-way ANOVA followed by Fisher’s LSD post hoc test. The correlation between water intake and urine amount was tested using Pearson’s correlation analyses. Results are presented for *n* = 6 rats in each experimental group.

## Results

### Diets

The sodium content was markedly higher in the Washed backbone diet and the Washed head diet when compared to the unwashed Backbone diet and the unwashed Head diet, as could be expected (Table 2).

**Table 2.** Dietary contents of sodium, amino acids, long-chain *n–*3 PUFA, and energy in the experimental diets

	Control diet	Backbone diet	Head diet	Washed backbone diet	Washed head diet
Sodium (mg/kg diet)	1200	1700	2100	3000	3400
Alanine (g/100 g diet)	0.60	0.83	0.81	0.84	0.85
Arginine (g/100 g diet)	0.69	0.88	0.89	0.93	0.93
Aspartic acid + asparagine (g/100 g diet)	1.4	1.5	1.5	1.6	1.6
Cysteine + cystine (g/100 g diet)	0.36	0.40	0.39	0.43	0.38
Glutamic acid + glutamine (g/100 g diet)	4.4	4.1	3.9	4.2	4.2
Glycine (g/100 g diet)	0.37	0.91	1.00	0.90	1.00
Histidine (g/100 g diet)	0.58	0.54	0.51	0.55	0.55
Isoleucin (g/100 g diet)	1.00	0.98	0.92	1.00	1.00
Leucine (g/100 g diet)	1.9	1.8	1.7	1.8	1.8
Lysine (g/100 g diet)	1.6	1.6	1.5	1.6	1.6
Methionine (g/100 g diet)	0.69	0.72	0.68	0.75	0.73
Phenylalanine (g/100 g diet)	1.00	0.97	0.92	1.00	1.00
Proline (g/100 g diet)	2.2	2.0	1.9	2.1	2.1
Hydroxyproline (g/100 g diet)	ND	0.2	0.2	0.1	0.2
Serine (g/100 g diet)	1.2	1.2	1.1	1.2	1.2
Threonine (g/100 g diet)	0.82	0.84	0.79	0.86	0.85
Tryptophan (g/100 g diet)	0.22	0.21	0.19	0.19	0.18
Tyrosine (g/100 g diet)	0.89	0.82	0.83	0.93	0.93
Valine (g/100 g diet)	1.3	1.2	1.1	1.3	1.2
EPA (mg/g diet)	ND	0.20	0.12	0.18	0.11
DHA (mg/g diet)	ND	0.32	0.23	0.31	0.25
Energy (kJ/g diet)	18.13	18.08	17.96	17.97	17.80

ND, not detected. Means of two measurements; deviations were <5% between parallels.

The diets containing backbone or head protein powder, either unwashed or washed with saltwater, differed marginally regarding the amino acid composition (Table 2). The percent differences from highest to lowest measured values of amino acids were ≤15% between diets containing cod proteins. The Control diet was characterised by lower contents of glycine, arginine, and alanine compared to diets containing cod protein powders. Hydroxyproline was found in all diets containing cod proteins but was not detected in the Control diet. The taurine content was very low in all diets; no taurine was detected in the Control diet, whereas the taurine content was around 15–20 mg per kg in the diets containing cod protein powders (data not presented).

EPA and DHA were not detected in the Control diet but were found in small amounts in the diets containing cod residuals (Table 2). The levels of both EPA and DHA were higher in the diets containing unwashed or saltwater-washed cod backbone proteins compared to diets containing Head protein powder; EPA was 39–40% higher, and DHA was 19–28% higher in Cod backbone diets. The dietary contents of other fatty acids were similar in the five diets and mainly reflected the contribution from soyabean oil that was added to all diets in a similar amount (Supplemental table 1).

**Figure 1.**
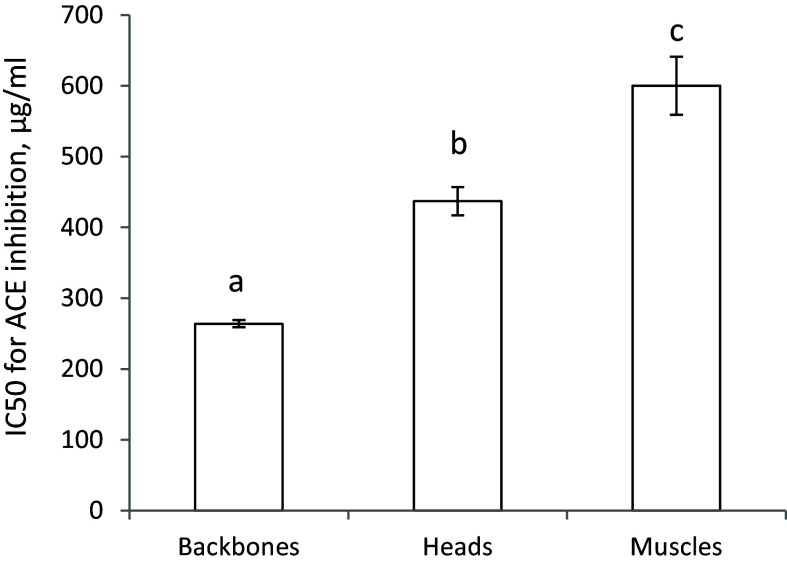
*In vitro* inhibition of activities of angiotensin-converting enzyme (ACE) by backbones, heads, and muscle protein powders. Values are means, with standard error of mean represented as vertical bars. Values are shown for two or three measurements, and means are compared using one-way ANOVA with LSD post hoc test. Bars with different letters are significantly different (*p* < 0.05).

The capacity for *in vitro* inhibition of ACE activity was significantly more potent for the backbone protein powder (that is, a lower IC50 concentration) compared to the head protein powder (Figure 1). For comparison, the inhibition of ACE activity by freeze dried cod muscle, which is more commonly used for human consumption, is also presented in in Figure 1, showing that cod muscle is less potent than both backbones and heads from cod.

### Dietary intake and bodyweight

The sodium intake was highest in rats fed the Washed head diet (Table 3). The water intake (g/kg bodyweight/24 h) and urine volume (g/kg bodyweight/24 h) were similar between the groups. Since both the water and urine measurements had relatively large SD, we checked if these were caused by measurement errors. However, the strong Pearson correlation between water intake and urine volume of 0.94 (*P* 3.1x10^−14^) suggests that the large variation between the individual rats were real.

**Table 3.** Bodyweight at baseline, bodyweight gain, kidney weight, dietary intake, water intake, urine volume and mean arterial pressure at baseline

	Control group	Backbone group	Head group	Washed backbone group	Washed head group	*p*
Sodium intake (mg/kg BW/24 h)	49 ± 6^a^	67 ± 12^ab^	89 ± 12^bc^	103 ± 32^c^	142 ± 21^d^	2.6 × 10^−7^
Water intake (g/kg BW/24 h)	60 ± 32	49 ± 27	51 ± 26	35 ± 11	45 ± 21	0.52
Urine volume (g/kg BW/24 h)	48 ± 35	41 ± 29	39 ± 24	29 ± 11	41 ± 22	0.78
BW (g) at baseline	361 ± 17	358 ± 10	356 ± 12	359 ± 14	356 ± 7	0.94
Weight gain (%)^1^	34 ± 6	35 ± 7	38 ± 4	37 ± 5	40 ± 7	0.39
Kidney weight (g/kg BW)	2.5 ± 0.2	2.4 ± 0.2	2.4 ± 0.2	2.4 ± 0.2	2.5 ± 0.2	0.94
Energy intake (kJ/kg BW/24 h)	739 ± 84	710 ± 126	764 ± 105	619 ± 189	794 ± 162	0.27
Protein intake (g/kg BW/24 h)	8.6 ± 1.0	8.5 ± 1.5	8.9 ± 1.2	7.7 ± 2.3	9.9 ± 2.0	0.27
MAP (mm Hg) at baseline	105 ± 9	107 ± 10	104 ± 14	112 ± 13	109 ± 11	0.79

Data are presented as mean ± standard deviation for *n* 6 rats in each dietary group. Groups were compared using one-way ANOVA and the *p* values in the table show results from the one-way ANOVA comparisons. Fisher’s LSD was used as post hoc test when appropriate, and different letters indicate significant differences between groups. *P* < 0.05 was considered significant. BW, bodyweight; MAP, mean arterial pressure.

1 Percent change in BW from baseline to endpoint.

The experimental groups had similar bodyweights at baseline, and the percent increase in bodyweight from baseline to endpoint was also similar between the groups (Table 3). The kidney weight (relative to bodyweight) was similar between the experimental groups. The daily intakes of energy and protein measured on day 30–31, after 24-h housing in metabolic cages, were similar between groups.

### Blood pressure

**Figure 2.**
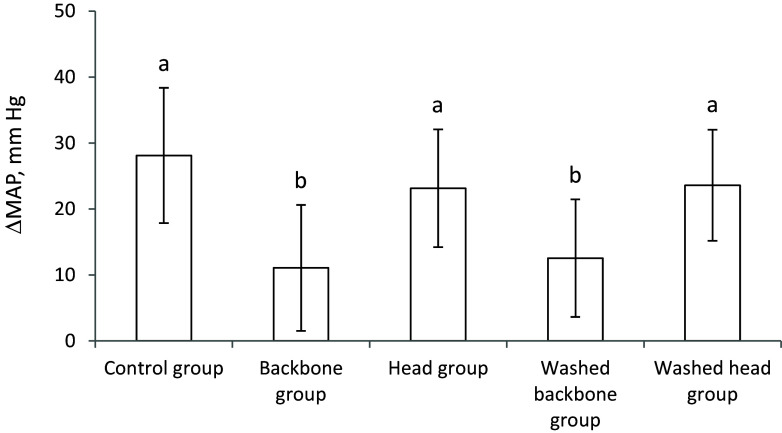
Changes in mean arterial blood pressure (MAP) from day 0 to day 40 are presented as mean with their standard deviation shown by vertical bars for *n* 6 rats in each group. Groups were compared using one-way ANOVA with Fisher’s LSD as post hoc test, and different letters indicate significant differences between groups. *p* < 0.05 was considered significant. MAP, mean arterial pressure.

The mean arterial blood pressure (MAP) was similar between the groups at baseline (Table 3). MAP was significantly increased from the baseline measurement to the endpoint measurements in all groups (*P* < 0.05), but the MAP was less increased in the unwashed Backbone group and in the Washed backbone group compared to the Control group, the unwashed Head group and the Washed head group (Figure 2). The rise in MAP was similar between the Backbone and the Washed backbone groups, and the increase in MAP was also similar between the Head group and the Washed head group.

### Angiotensin-converting enzyme

The concentration and activity of ACE in serum were similar between the groups, and no difference between the groups was seen for lung ACE concentration (relative to total protein) (Table 4). The renal ACE concentration was higher in the group fed the Washed head diet when compared to the other experimental groups, whereas the groups fed the unwashed Backbone diet, the unwashed Head diet or the Washed backbone diet had similar renal ACE concentration to the Control group. The groups fed the unwashed or washed backbone diets had a lower renal ACE concentration compared to rats fed the unwashed Head diet or the Washed head diet.

**Table 4.** Angiotensin-converting enzyme (ACE) in serum, kidneys, and lungs

	Control group	Backbone group	Head group	Washed backbone group	Washed head group	*p*
Serum ACE (ng/ml)	220 ± 128	138 ± 84	196 ± 157	198 ± 140	179 ± 108	0.84
Lung ACE (ng/mg protein)	6.85 ± 2.20	6.71 ± 2.02	6.64 ± 1.59	5.88 ± 0.90	7.20 ± 1.07	0.72
Renal ACE (ng/mg protein)	685 ± 111^ab^	584 ± 113^a^	733 ± 110^b^	577 ± 71^a^	966 ± 99^c^	1.0 × 10^−5^
Serum ACE activity, FRKP both domains (RFU/min)	1336 ± 179	1280 ± 112	1427 ± 256	1395 ± 153	1354 ± 131	0.64
Serum ACE activity, LFK - C-domain (RFU/min)	32.0 ± 6.5	29.1 ± 3.3	32.7 ± 9.8	33.5 ± 7.2	34.9 ± 7.1	0.70
Serum ACE activity, SDKP - N-domain (RFU/min)	10.9 ± 1.3	9.8 ± 1.5	11.0 ± 1.8	10.6 ± 1.2	10.7 ± 1.2	0.58

Data are presented as mean ± standard deviation for *n* 6 rats in each dietary group. Groups were compared using one-way ANOVA and the *p* values in the table show results from the one-way ANOVA comparisons. Fisher’s LSD was used as post hoc test when appropriate, and different letters indicate significant differences between groups. *p* < 0.05 was considered significant.

### Markers of kidney function and taurine in serum

The serum concentrations of alanine transaminase, aspartate transaminase, NT-proBNP and taurine were similar between all dietary groups at endpoint (Table 5). The groups were also similar with regard to the urine concentrations (relative to creatinine) of cystatin C, TIM-1, albumin, carbamide, uric acid and nitrate + nitrite.

**Table 5.** Markers of kidney function and organ damage, serum taurine concentration, and urine concentration (relative to creatinine) of nitrite+nitrate

	Control group	Backbone group	Head group	Washed backbone group	Washed head group	*p*
Serum alanine transaminase (U/L)	146 ± 53	121 ± 55	164 ± 85	164 ± 75	125 ± 43	0.66
Serum aspartate transaminase (U/L)	286 ± 159	208 ± 125	294 ± 194	297 ± 186	251 ± 123	0.85
Serum NT-proBNP (ng/ml)	19.9 ± 5.7	16.0 ± 4.3	19.0 ± 4.3	19.1 ± 1.1	17.2 ± 4.3	0.49
Serum taurine (μmol/L)	128 ± 17	128 ± 13	128 ± 10	122 ± 8	131 ± 14	0.82
Urine cystatin C (µg/mmol creatinine)	2177 ± 1258	2231 ± 1082	1779 ± 649	1836 ± 729	1949 ± 746	0.89
Urine TIM-1 (ng/mmol creatinine)	418 ± 238	305 ± 157	225 ± 83	238 ± 84	321 ± 243	0.34
Urine albumin (µg/mmol creatinine)	6.3 ± 6.4	3.2 ± 1.7	4.1 ± 2.8	4.5 ± 2.0	7.4 ± 5.2	0.45
Urine carbamide (mmol/mmol creatinine)	329 ± 90	351 ± 99	353 ± 93	294 ± 56	383 ± 68	0.46
Urine uric acid (μmol/mmol creatinine)	243 ± 61	276 ± 105	266 ± 106	341 ± 74	296 ± 71	0.59
Urine nitrite + nitrate (µmol/mmol creatinine)	120 ± 54	120 ± 59	116 ± 44	146 ± 31	141 ± 31	0.71

Data are presented as mean ± standard deviation for *n* 6 rats in each dietary group. Groups were compared using one-way ANOVA, and the *p* values in the table show results from the one-way ANOVA comparisons. No post hoc tests were conducted since the ANOVA showed no between-group differences. *p* < 0.05 was considered significant.

ACE, angiotensin-converting enzyme; NT-proBNP, N-terminal Prohormone Brain Natriuretic Peptide; TIM-1, T cell immunoglobulin mucin-1.

### Sodium excretion and storage

The 24-h sodium excretion in urine was significantly higher in rats fed the diet containing cod heads washed with saltwater when compared to all other groups (Table 6). The sodium contents in skin, aorta, and heart muscle were similar between the groups.

**Table 6.** Sodium excretion in urine (24 h) and sodium concentration in skin, heart muscle, and aorta

	Control group	Backbone group	Head group	Washed backbone group	Washed head group	*p*
Sodium in urine (mmol/24 h)	1.03 ± 1.10^a^	1.29 ± 1.01^a^	1.23 ± 1.10^a^	1.54 ± 0.38^a^	2.97 ± 1.35^b^	0.025
Sodium in skin (mmol/g)	32.3 ± 5.7	31.3 ± 4.7	30.4 ± 2.8	30.3 ± 4.0	30.2 ± 3.9	0.89
Sodium in heart muscle (mmol/g)	35.5 ± 2.4	35.3 ± 2.2	37.5 ± 2.2	37.6 ± 1.1	35.8 ± 2.9	0.24
Sodium in aorta (mmol/g)	68.3 ± 3.9	70.6 ± 6.2	69.8 ± 4.8	68.0 ± 4.1	68.5 ± 2.4	0.82

Data are presented as mean ± standard deviation for *n* 6 rats in each dietary group. Groups were compared using one-way ANOVA, and the *p* values in the table show results from the one-way ANOVA comparisons. Fisher’s LSD was used as post hoc test when appropriate, and different letters indicate significant differences between groups. *p* < 0.05 was considered significant.

## Discussion

This study is the first to present evidence that cod backbone protein powders attenuate the development of high blood pressure in obese Zucker *fa/fa* rats, and this effect was not counteracted when the sodium content in the backbone protein powder was elevated after saltwater washing. Protein powders produced from cod heads did not affect the blood pressure development in this rat model, neither as unwashed nor saltwater-washed powders.

High blood pressure is the leading modifiable risk factor for CVD morbidity and mortality globally,^(48, 49)^ and a healthy low-salt diet is recommended.^(3–5)^ Dietary intake of fish fillet is associated with lower blood pressure in humans,^(8–13)^ however, little is known about whether consumption of fish residual proteins will affect blood pressure. Adding value to fish byproducts through sustainable fish processing of high-quality products for human consumption is in line with the United Nations Sustainable Development Goal 12 for responsible consumption and production, and increased use of fish residuals for fishmeal production will have a beneficial impact on waste reduction. In order to obtain high-quality fishmeal with increased shelf life and improved sensory properties, the residuals may be washed with water to remove substances such as haemoglobin containing iron (which induces oxidation) and trimethylamine (which gives an unpleasant fishy smell). Freshwater is a limited resource on factory trawlers with on-board processing, and seawater is a more available and sustainable choice for this washing process. As expected, washing with saltwater increased the sodium content in the residual protein powders, thus resulting in a higher sodium intake in rats consuming the saltwater-washed residual protein powders from cod backbones or heads in the present study.

In this study, we used male obese Zucker *fa/fa* rats that were 8–9 weeks old when the intervention started, which is around the age when the blood pressure spontaneously starts to increase and the development of proteinuria and focal segmental glomerulosclerosis begins to emerge in this rat model.^(14, 32, 50, 51)^ The obese Zucker *fa/fa* rats are salt-sensitive and will experience increased blood pressure when fed a diet with high sodium content.^(31)^ The addition of saltwater-washed backbone and head protein fractions to the present diets resulted in sodium contents of 0.30 and 0.34 wt% in the Washed backbone diet and the Washed head diet, respectively, which were higher compared to diets containing unwashed backbone or head protein fractions and the Control diet. In general, rat diets are considered as high salt diets when the sodium content is above 2–3 wt% (depending on the definition). The sodium storage in tissues was not affected by any of the diets containing unwashed or washed cod residuals, but the highest sodium intake in the Washed head group was accompanied by the highest urinary sodium excretion. Based on the present study, a sodium content of around 3 wt‰ from cod residuals did not adversely affect the blood pressure development in obese Zucker *fa/fa* rats.

The renin-angiotensin-aldosterone system is important for the control of blood pressure and includes the conversion of angiotensinogen via angiotensin I to the vasoconstrictor angiotensin II catalysed by several enzymes including ACE.^(21)^ Therefore, inhibiting ACE may lead to a lower blood pressure through reduced production of angiotensin II. Proteins found in fish muscles or residuals contain a plethora of amino acid sequences that have been shown to have ACE inhibiting activities,^(26–29)^ and direct *in vitro* measurements have demonstrated that proteins from muscles, viscera, skin and other residuals from several fish species including cod can inhibit ACE activity.^(16, 17, 19, 28, 29, 52, 53)^ In the present study, the Backbone protein powder was almost twice as potent as Head protein powder as an inhibitor of ACE activity *in vitro*. This may, at least in part, explain the attenuation of blood pressure increase after consuming diets containing backbones, whereas the head-containing diets had no effect on blood pressure development but was similar to the Control group. To investigate further, the concentration and activity of ACE were measured in serum, and ACE protein content was quantified in lungs and kidneys since these are major organs for ACE synthesis. The renal ACE protein concentration was highest in the group fed the Washed head diet, and it is of interest that this group also had the highest sodium intake. This is in line with reports showing higher gene expression of ACE in kidneys from Zucker *fa/fa* rats fed a high salt diet.^(54)^ In contrast, we found no differences between the experimental groups for serum and pulmonary ACE. From the present findings it is difficult to conclude whether the effect on blood pressure after cod backbone intake involves the ACE pathway, but the strong *in vitro* ACE inhibition by Backbone protein powder makes it compelling to suggest that a direct inhibition of ACE activity as a plausible, and at least a partial, explanation for the observed attenuation of the blood pressure increase after Backbone protein intake in this rat model. This finding corresponds well with our previous study investigating the effects of a range of cod residual protein powders in rats, where the most efficient residual fraction on blood pressure attenuation in obese Zucker *fa/fa* rats also was the most potent inhibitor of ACE activity in vitro.^(16)^

The difference in the blood pressure-delaying effects of diets containing unwashed or saltwater-washed backbone proteins or head proteins cannot be explained by differences in sodium content. However, the identification of the nutrient(s) responsible for the beneficial delay in blood pressure increase after consuming backbone protein powders but not after head protein powder is not straightforward. Several nutrients and other compounds in foods may affect blood pressure, and we have chosen to quantify some of the most central compounds in the rat feeds. Arginine is of interest since arginine supplementation has been shown to lower blood pressure in humans.^(55)^ Arginine is a substrate for vascular production of the vasodilator nitric oxide,^(56)^ and arginine in fish is proposed to be a contributor to the delayed development of hypertension of dietary fish proteins fed to spontaneously hypertensive rats.^(57)^ The arginine content was similar between the diets containing backbones or heads and was only marginally higher in the cod-containing diets when compared to the Control diet. In addition, the urine nitrite + nitrate concentration (as a measure of nitric oxide, synthesised from arginine) was similar between all groups. This strongly indicated that dietary arginine was not responsible for the attenuated blood pressure increase in rats fed diets containing unwashed or washed backbone proteins. Taurine, either from the diet or endogenously produced from methionine via cysteine, is also of interest as taurine may lower blood pressure by antagonising the effects of angiotensin II activity and thus suppressing the renin-angiotensin-aldosterone system.^(58)^ The taurine content in the cod-containing diets was comparable and very low, and the taurine content in the Control diet was below the detection level. In addition, the serum taurine concentration was similar between all experimental groups. Hence, it is not likely that the observed effects on the blood pressure after consumption of diets containing unwashed or washed backbone protein powders were related to taurine intake or endogenous taurine production. Other candidates as blood pressure-lowering nutrients in the diets include the long-chain *n*−3 PUFAs, and studies in humans^(9, 59, 60)^ and rats^(15, 61, 62)^ have demonstrated an inverse association between fatty fish or fish oil intake and blood pressure. In the present study, both EPA and DHA contents were higher in the diets containing backbones compared to diets containing heads, and although the dietary contents of these fatty acids were low, dietary EPA and DHA may have contributed to the beneficial effect of backbone proteins on blood pressure development.

A reduced kidney function may contribute to a higher blood pressure through failure to remove waste, sodium, and excess fluid, and vice versa, hypertension can impair the kidney function. The obese Zucker *fa/fa* rat develops proteinuria when it is about ten weeks old^(32)^ with signs of podocyte damage due to increased glomerular intravascular pressure.^(63)^ In this study, we used obese Zucker *fa/fa* rats that were 8–9 weeks old at the start of the 6-week intervention period, thus the kidney function was expected to be aggravated during the intervention period. All rats had high urine concentrations of cystatin C and TIM-1, which are specific markers of tubular dysfunction or injury,^(64, 65)^ with no differences between the groups. This, and the similar urine concentrations of albumin, carbamide and uric acid as well as the similar kidney weight between the groups, indicate that neither of the diets containing backbone or head protein powder, regardless of the dietary sodium content, delayed the development of kidney damage. This corresponds well with our recent report where a mixture of heads, backbones and skin did not protect young obese Zucker *fa/fa* rats against kidney dysfunction.^(20)^ Despite the aggravated renal function in all rats at the end of the experiment, the excretion of sodium over the kidneys was sufficient to prevent excess accumulation of sodium in tissues. The attenuation of blood pressure increase by diets containing backbone proteins did not affect the serum concentration of NT-proBNP, which is a vasodilator and has natriuretic effects, indicating no effect by the fish protein on this indicator of heart failure. In addition, the serum concentrations of alanine transaminase and aspartate transaminase, which are important markers for organ damage including fatty liver, were similar between all groups. This indicates that diets containing unwashed or washed backbone or head protein powders from cod did not protect the obese Zucker *fa/fa* against the development of kidney failure or fatty liver, and that the higher sodium content in the saltwater-washed residuals did not adversely affect organ functions in these rats.

The present study has some methodological strengths and limitations. We used obese Zucker *fa/fa* rats which develop an age-related increase in blood pressure resembling what is seen in humans,^(66)^ since this is a relevant model where hypertension co-exists with obesity and other metabolic disturbances including salt sensitivity.^(31, 51)^ We consider this model to be more relevant than the more commonly used spontaneously hypertensive rat, which is representative of a rare subtype of human hypertension, that is, primary hypertension that is inherited in a Mendelian fashion,^(67)^ demonstrating only a small segment of the aetiologies of hypertension. All blood pressure measurements were conducted in conscious rats using the tail-cuff method (volume-pressure recording), and the rats were hand-tamed and trained to be in the constrainer. The tail-cuff method is non-invasive and inexpensive and was chosen instead of continuous intravascular blood pressure measured by telemetry as the comparison of these methods shows similar results over the physiological range of blood pressure.^(68)^ A limitation to the study is that we tested only two types of residual materials, and we did not test fish species other than Atlantic cod. Thus, the findings from the present study cannot be generalised to other fish species or other types of residuals. Although the study is limited by a relatively small sample size, the direction and consistency of the observed effect of unwashed and saltwater-washed head proteins vs. unwashed and saltwater-washed backbone proteins on the blood pressure development strengthen the conclusion. The small sample size is a limitation to the statistical power of this study and weakens the robustness of the overall findings and thus the conclusion. To examine if the effect of cod residual protein powders on blood pressure, the ACE inhibitory potential of the cod protein fractions was investigated using an *in vitro* assay, and although this does not provide evidence for the mechanism of action behind the observed effects on the blood pressure, we still consider this to be of interest. The quantification of ACE protein concentrations in tissues can be regarded as more relevant compared to measuring ACE mRNA expression, but measurements of ACE activity in the relevant organs would be even better, and we aim to establish methods for measurements of ACE activity in organs in future studies, taking fresh samples from rats fed fish byproducts.

The sodium content in the saltwater-washed residuals used in the present study is relevant for future human fish protein powders that are produced onboard factory trawlers where haemoglobin and trimethylamine are removed using seawater to achieve longer shelf life and better sensory properties. Utilising more of the proteins from fish residuals for human consumption will increase the sustainability of fisheries and provides a source for high-quality protein. The findings in the present study may have relevance for the future development and use of fish residuals, as the small increase in sodium after saltwater washing did not adversely affect blood pressure in the obese and salt-sensitive Zucker *fa/fa* rat. Our data indicate that using seawater to wash residuals to increase shelf life and improve sensory properties is safe, and future studies should investigate this further in humans with increased risk of developing hypertension due to obesity or salt sensitivity.

## Conclusion

In the present study we present evidence that saltwater-washed cod residuals did not adversely affect blood pressure development when fed to obese Zucker *fa/fa* rats which spontaneously develop hypertension, despite the higher sodium content. An attenuation of the blood pressure increase was seen after consumption of unwashed and saltwater-washed backbone protein powders, whereas the head protein powders did not affect blood pressure development when compared to the Control diet. This aligns with the finding that the backbone protein powder was almost twice as potent as the head protein powder as an inhibitor of ACE activity *in vitro*.

## Supporting information

10.1017/jns.2026.10099.sm001 Rimmen et al. supplementary material Rimmen et al. supplementary material

## Supplementary material

The supplementary material for this article can be found at https://doi.org/10.1017/jns.2026.10099.
