# Supplementary material for: Increased sodium content after saltwater washing did not compromise the attenuation of blood pressure increase by cod backbone proteins in obese Zucker fa/fa rats
Source: J Nutr Sci. 2026 May 13;15:e33. doi: 10.1017/jns.2026.10099 (PMC13168912; doi:10.1017/jns.2026.10099)
Supplement: Rimmen et al. supplementary material [file S2048679026100998sup001.docx]

**Supplemental table:** Dietary contents of fatty acids

|  | Control diet | Backbone diet | Head diet | Washed backbone diet | Washed head diet |
| --- | --- | --- | --- | --- | --- |
| Fatty acids (mg/g diet) |  |  |  |  |  |
| 14:0 | 0.25 | 0.26 | 0.25 | 0.24 | 0.25 |
| 16:0 | 6.79 | 7.12 | 7.12 | 6.97 | 7.15 |
| 17:0 | 0.07 | 0.07 | 0.07 | 0.08 | 0.07 |
| 18:0 | 2.24 | 2.32 | 2.35 | 2.29 | 2.35 |
| 20:0 | 0.16 | 0.16 | 0.16 | 0.16 | 0.16 |
| 22:0 | 0.17 | 0.17 | 0.19 | 0.19 | 0.18 |
| 16:1 n-7 | 0.08 | 0.12 | 0.11 | 0.12 | 0.11 |
| 18:1 n-9 | 12.09 | 12.42 | 12.54 | 12.23 | 12.61 |
| 18:1 n-7 | 0.83 | 0.87 | 0.89 | 0.89 | 0.91 |
| 20:1 n-9 | 0.09 | 0.17 | 0.17 | 0.17 | 0.15 |
| 18:2 n-6 | 31.67 | 32.07 | 32.45 | 31.69 | 32.46 |
| 18:3 n-3 | 4.02 | 4.06 | 4.11 | 4.03 | 4.10 |
| 20:5 n-3 | ND | 0.20 | 0.12 | 0.18 | 0.11 |
| 22:6 n-3 | ND | 0.32 | 0.23 | 0.31 | 0.25 |

ND: not detected.
